# Cutaneous Manifestations of SARS-CoV-2, Cutaneous Adverse Reactions to Vaccines Anti-SARS-CoV-2 and Clinical/Dermoscopical Findings: Where We Are and Where We Will Go

**DOI:** 10.3390/vaccines11010152

**Published:** 2023-01-10

**Authors:** Gerardo Cazzato

**Affiliations:** Section of Molecular Pathology, Department of Precision and Regenerative Medicine and Ionian Area (DiMePRe-J), University of Bari Aldo Moro, 70124 Bari, Italy; gerardo.cazzato@uniba.it

From the very first months of the pandemic, it became apparent that a variety of skin reactions could occur during COVID-19 disease, starting with ‘erythema-pernio’-type lesions, similar to chilblains [1,2], up to more extensive eruptions, including erythema morbilliforme/maculo-papular lesions [3], urticarial lesions [4], petechial/purpuric lesions [5,6,7,8], varicelliform exanthema [9] and many others [10]. Over the months, a growing body of scientific evidence has attempted to study the complex relationship between SARS-CoV-2 and the skin; in particular, various types of analysis have been conducted with the ultimate aim of understanding whether it was the virion that penetrated the various structures of the skin (with particular attention to the excretory portion of the eccrine sweat glands) [11,12] or whether the infection itself determined an immune-mediated mechanism that, as a ‘secondary’ effect, induced skin manifestations of the most diverse types [13]. Our group, at the beginning of 2021, reported a case history of 17 cases of patients with various skin manifestations, of which 7 with erythema Multiforme-like lesions, 7 with pseudochildblains, 2 patients with Chickenpox rash and 1 patient with urticarioid rash. The skin biopsies of these subjects were subjected to immunostaining with anti-SARS-CoV-2 spike S1 glycoprotein monoclonal antibody, Thermofisher, Rabbit, at pH 6, diluted 1:800 and the results were analyzed semi-quantitatively, showing positivity of the brown signal in the excretory portion of the eccrine sweat glands and the vascular endothelium [11]. In addition, the biopsies were subjected to PCR with positive and confirmatory results. However, subsequent studies in the literature did not always describe these features. For instance, Ko et al. [13] addressed the problem of poor concordance between immunostaining anti-Spike protein of SARS-CoV-2 and PCR or RNA in situ Hybridization (ISH). In their work, in fact, the authors suggest the far from a remote possibility that rather than entire virions of SARS-CoV-2, there are clipped fragments of Spike protein that binds via expression of angiotensin-converting enzyme receptor-2 (ACE2-R) to the endothelium of blood vessels and the excretory portion of eccrine sweat glands. However, we do not yet have an unambiguous answer [14], although we consider that both mechanisms (direct virion penetration and indirect cytokines/chemokines) are likely to play a role in the determinism of such rashes. To date, various models have been used to explain the pathogenesis of these manifestations; for instance, maculopapular and urticarial eruptions are mainly believed to be due to an adverse reaction to COVID-19 pharmaceutical drugs or to cytokine overproduction triggered by hyperinflammation [15]. On the other hand, possible etiopathogenesis mechanisms underlying chilblain-like lesions are quite varied, including immunological dysregulation, vasculitis, vascular thrombosis or neoangiogenesis [16]. Finally, petechial/purpuric lesions appear to involve a thrombogenic pauci-inflammatory vasculopathy with extensive deposition of C5b-9 and C4d complement components within the skin microvasculature [10].

The advent of anti-SARS-CoV-2 vaccines has been a milestone in the battle against ongoing COVID-19 morbidity and mortality, as well as playing a leading role in the possibility of reopening and relaxing worldwide restrictions [17]. Since the early months of the vaccination campaign, an increasing number of views has been more or less equally reflected in the study and analysis of ongoing skin manifestations of the anti-SARS-CoV-2 vaccine, both of the more traditional adenovirus vectors and mRNA vaccines, such as Pfizer-BioNTech’s BNT162b2 vaccine and Moderna’s mRNA-1273 vaccine. In particular, after the first few months of the advent of vaccination campaigns, an increasing number of adverse reactions after vaccination began to be recorded and, although initially small, studies with histopathological correlations were also published [18,19,20,21]. The diatribe as to whether it was SARS-CoV-2 that penetrated the skin rather than an immune reaction linked to COVID-19 has also arisen in this field: what is the real cause of adverse reactions to vaccines? Vaccine adjuvants, such as polyethylene glycol (PEG) [22], lipid nanoparticles [23] or a subunit reaction to the SARS-CoV-2 spike protein for which the vaccine mRNA encodes?

The research does not yet have a definitive answer: the various studies conducted on the subject have yielded rather conflicting and, in some respects, opposing results. For instance, some works have suggested that the main mechanism of pathogenesis of cutaneous adverse reactions (both at the injection site and generalized) is a delayed type IV hypersensitivity reaction, potentially directed towards mRNA-based vaccine components not present in other preparations, such as the Johnson & Johnson vaccine. Magro C. el al. [24] extensively analyzed how post-vaccine reactions tend to occur after both the first and second dose, and that a reaction to the first dose appears to have a high positive predictive value for a second reaction to the second vaccine administration. Similarly, it appears that women are more prone to develop reactions to the vaccine and, incidentally, it would appear from the paper by McMahon et al. [25] that 80% of ADRs occurred with the Moderna vaccine. As noted by Magro C., the most frequent histopathological patterns are systemic hypersensitivity reactions of the eczematoid type. Indeed, the most common histopathological picture is that of a type IV hypersensitivity, characterized by eczematous dermatitis and/or concomitant cytotoxic interface dermatitis. This finding would suggest that the antigen could be a substance contained in the vehicle used to administer the vaccine, although a T-cell and/or humoral reaction to the spike glycoprotein produced by myocytes emerges as a presumed antigenic trigger, especially in view of its localization in the skin microvessels [26].

In view of the above, research on these issues is still in full swing and only new work with large case series and a focus on these aspects will be able to help elucidate the pathogenetic mechanisms responsible for these manifestations, with particular attention on the role that confirmatory biopsy plays in guiding our knowledge of the type of immune response involved.

## References

[1] Wang C.J., Worswick S. (2021). Cutaneous manifestations of COVID-19. Dermatol. Online J..

[2] Ingravallo G., Mazzotta F., Resta L., Sablone S., Cazzato G., Cimmino A., Rossi R., Colagrande A., Ferrante B., Troccoli T. (2021). Inflammatory Skin Lesions in Three SARS-CoV-2 Swab-Negative Adolescents: A Possible COVID-19 Sneaky Manifestation?. Pediatr. Rep..

[3] Galván Casas C., Català A., Hernández G.C. (2020). Classification of the cutaneous manifestations of COVID-19: A rapid prospective nationwide consensus study in Spain with 375 cases. Br. J. Dermatol..

[4] Van Damme C., Berlingin E., Saussez S., Accaputo O. (2020). Acute urticaria with pyrexia as the first manifestations of a COVID-19 infection. J. Eur. Acad. Dermatol. Venereol..

[5] de Masson A., Bouaziz J.D., Sulimovic L., Cassius C., Jachiet M., Ionescu M.A., Rybojad M., Bagot M., Duong T.A., SNDV (French National Union of Dermatologists-Venereologists) (2020). Chilblains is a common cutaneous finding during the COVID-19 pandemic: A retrospective nationwide study from France. J. Am. Acad. Dermatol..

[6] Askin O., Altunkalem R.N., Altinisik D.D., Uzuncakmak T.K., Tursen U., Kutlubay Z. (2020). Cutaneous manifestations in hospitalized patients diagnosed as COVID-19. Dermatol. Ther..

[7] Rubio-Muniz C.A., Puerta-Peña M., Falkenhain-López D., Arroyo-Andrés J., Agud-Dios M., Rodriguez-Peralto J.L., Ortiz-Romero P.L., Rivera-Díaz R. (2020). The broad spectrum of dermatological manifestations in COVID-19: Clinical and histopathological features learned from a series of 34 cases. J. Eur. Acad. Dermatol. Venereol..

[8] De Giorgi V., Recalcati S., Jia Z., Chong W., Ding R., Deng Y., Scarfi F., Venturi F., Trane L., Gori A. (2020). Cutaneous manifestations related to coronavirus disease 2019 (COVID-19): A prospective study from China and Italy. J. Am. Acad. Dermatol..

[9] Gaspari V., Neri I., Misciali C., Patrizi A. (2020). COVID-19: How it can look on the skin. Clinical and pathological features in 20 COVID-19 patients observed in Bologna, north-eastern Italy. J. Eur. Acad. Dermatol. Venereol..

[10] Singh H., Kaur H., Singh K., Sen C.K. (2021). Cutaneous Manifestations of COVID-19: A Systematic Review. Adv. Wound Care.

[11] Cazzato G., Mazzia G., Cimmino A., Colagrande A., Sablone S., Lettini T., Rossi R., Santarella N., Elia R., Nacchiero E. (2021). SARS-CoV-2 and Skin: The Pathologist’s Point of View. Biomolecules.

[12] Colmenero I., Santonja C., Alonso-Riaño M., Noguera-Morel L., Hernández-Martín A., Andina D., Wiesner T., Rodríguez-Peralto J.L., Requena L., Torrelo A. (2020). SARS-CoV-2 endothelial infection causes COVID-19 chilblains: Histopathological, immunohistochemical and ultrastructural study of seven paediatric cases. Br. J. Dermatol..

[13] Ko C.J., Harigopal M., Gehlhausen J.R., Bosenberg M., McNiff J.M., Damsky W. (2021). Discordant anti-SARS-CoV-2 spike protein and RNA staining in cutaneous perniotic lesions suggests endothelial deposition of cleaved spike protein. J. Cutan. Pathol..

[14] Cappel M.A., Cappel J.A., Wetter D.A. (2021). Pernio (Chilblains), SARS-CoV-2, and COVID Toes Unified Through Cutaneous and Systemic Mechanisms. Mayo Clin. Proc..

[15] Türsen Ü., Türsen B., Lotti T. (2020). Cutaneous side-effects of the potential COVID-19 drugs. Dermatol. Ther..

[16] Bouaziz J.D., Duong T.A., Jachiet M., Velter C., Lestang P., Cassius C., Arsouze A., Trong E.D.T., Bagot M., Begon E. (2020). Vascular skin symptoms in COVID-19: A French observational study. J. Eur. Acad. Dermatol. Venereol..

[17] Tregoning J.S., Flight K.E., Higham S.L., Wang Z., Pierce B.F. (2021). Progress of the COVID-19 vaccine effort: Viruses, vaccines and variants versus efficacy, effectiveness and escape. Nat. Rev. Immunol..

[18] Sun Q., Fathy R., McMahon D.E., Freeman E.E. (2021). COVID-19 Vaccines and the Skin: The Landscape of Cutaneous Vaccine Reactions Worldwide. Dermatol. Clin..

[19] Gambichler T., Boms S., Susok L., Dickel H., Finis C., Rached N.A., Barras M., Stücker M., Kasakovski D. (2022). Cutaneous findings following COVID-19 vaccination: Review of world literature and own experience. J. Eur. Acad. Dermatol. Venereol..

[20] Ambrogio F., Laface C., Sbarra G., Filotico R., Ranieri G., Barlusconi C., De Marco A., Cazzato G., Bonamonte D., Romita P. (2022). A Case of Purpura Annularis Telangiectodes of Majocchi after Anti-SARS-CoV-2 Pfizer-BioNTech Vaccine: Is There an Association?. Vaccines.

[21] Cazzato G., Romita P., Foti C., Lobreglio D., Trilli I., Colagrande A., Ingravallo G., Resta L. (2022). Development of Flat Warts on the Cheeks after BioNTech-Pfizer BNT162b2 Vaccine: Is There a Correlation?. Vaccines.

[22] Rutkowski K., Wagner A., Rutkowski R. (2020). Immediate hypersensitivity reactions to steroids and steroid containing medications. Curr. Opin. Allergy Clin. Immunol..

[23] Alameh M.G., Tombácz I., Bettini E., Lederer K., Sittplangkoon C., Wilmore J.R., Gaudette B.T., Soliman O.Y., Pine M., Hicks P. (2022). Lipid nanoparticles enhance the efficacy of mRNA and protein subunit vaccines by inducing robust T follicular helper cell and humoral responses. Immunity.

[24] Magro C., Crowson A.N., Franks L., Schaffer P.R., Whelan P., Nuovo G. (2021). The histologic and molecular correlates of COVID-19 vaccine-induced changes in the skin. Clin. Dermatol..

[25] McMahon D.E., Amerson E., Rosenbach M., Lipoff J.B., Moustafa D., Tyagi A., Desai S.R., French L.E., Lim H.W., Thiers B.H. (2021). Cutaneous reactions reported after Moderna and Pfizer COVID-19 vaccination: A registry-based study of 414 cases. J. Am. Acad. Dermatol..

[26] Restivo V., Candore G., Barrale M., Caravello E., Graziano G., Onida R., Raineri M., Tiralongo S., Brusca I. (2021). Allergy to Polyethilenglicole of Anti-SARS CoV2 Vaccine Recipient: A Case Report of Young Adult Recipient and the Management of Future Exposure to SARS-CoV2. Vaccines.

